# Investigating the causal effects of childhood and adulthood adiposity on later life mental health outcome: a Mendelian randomization study

**DOI:** 10.1186/s12916-024-03765-6

**Published:** 2025-01-06

**Authors:** Sweta Pathak, Tom G. Richardson, Eleanor Sanderson, Bjørn Olav Åsvold, Laxmi Bhatta, Ben M. Brumpton

**Affiliations:** 1 HUNT Center for Molecular and Clinical Epidemiology, Department of Public Health and Nursing, Norwegian University of Science and Technology, Trondheim, Norway; 2 MRC Integrative Epidemiology Unit, Population Health Sciences, Bristol Medical School, University of Bristol, Bristol, UK; 3 Department of Endocrinology, Clinic of Medicine, St. Olavs Hospital, Trondheim University Hospital, 7030 Trondheim, Norway; 4 HUNT Research Centre, Department of Public Health and Nursing, NTNU Norwegian University of Science and Technology, Levanger, Norway; 5 Clinic of Medicine, St. Olavs Hospital, Trondheim University Hospital, Trondheim, Norway

**Keywords:** Childhood, Adulthood, Obesity, Depression, Anxiety, Mendelian randomization

## Abstract

**Background:**

Obesity particularly during childhood is considered a global public health crisis and has been linked with later life health consequences including mental health. However, there is lack of causal understanding if childhood body size has a direct effect on mental health or has an indirect effect after accounting for adulthood body size.

**Methods:**

Two-sample Mendelian randomization (MR) was performed to estimate the total effect and direct effect (accounting for adulthood body size) of childhood body size on anxiety and depression. We used summary statistics from a genome-wide association study (GWAS) of UK Biobank (*n* = 453,169) and large-scale consortia of anxiety (Million Veteran Program) and depression (Psychiatric Genomics Consortium) (*n* = 175,163 and *n* = 173,005, respectively).

**Results:**

Univariable MR did not indicate genetically predicted effects of childhood body size with later life anxiety (beta = − 0.05, 95% CI = − 0.13, 0.02) and depression (OR = 1.06, 95% CI = 0.94, 1.20). However, using multivariable MR, we observed that the higher body size in childhood reduced the risk of later life anxiety (beta = − 0.19, 95% CI = − 0.29, − 0.08) and depression (OR = 0.83, 95% CI = 0.71, 0.97) upon accounting for the effect of adulthood body size. Both univariable and multivariable MR indicated that higher body size in adulthood increased the risk of later life anxiety and depression.

**Conclusions:**

Higher body size in adulthood may increase the risk of anxiety and depression, independent of childhood higher body size. In contrast, higher childhood body size does not appear to be a risk factor for later life anxiety and depression.

**Supplementary Information:**

The online version contains supplementary material available at 10.1186/s12916-024-03765-6.

## Background

Obesity is a global pandemic with a prevalence that has tripled since 1975 and it is a leading risk factor for adverse health outcomes including cardiometabolic diseases [1]. Obesity particularly during childhood is considered a global public health crisis and has increased tenfold since 1975 [1, 2]. Childhood obesity has been linked to several health outcomes including cardiometabolic diseases, premature mortality, and mental disorders [1, 3–10].

According to World Health Organization in 2019, 1 in every 8 people were living with mental disorder among which anxiety (301 million) and depression (280 million) are most common and are frequently comorbid with one another [11–13]. Several observational and genetic studies have reported associations between higher adult adiposity and anxiety and depression [14–18]. The associations between childhood adiposity and childhood anxiety and depression [7, 19, 20], and childhood adiposity and adulthood anxiety and depression are less clear [21]. If childhood adiposity increases both childhood and adulthood anxiety and depression, it may be an important time point for prevention strategies. However, except for these observational studies, no genetic studies have been performed to investigate the time-specific effect of childhood adiposity on later life anxiety and depression.

Simmonds et al. observed that around 55% of children with obesity went on to be obese in adolescence, meaning nearly half were only obese during their childhood [22]. Also, there is a moderate genetic correlation (rg = 0.61) between childhood and adulthood body size [23]. Thus, identifying the long-term effects of childhood adiposity requires methods that disentangle the association of childhood and adulthood body size. Several reports suggest that the early years of life play a crucial role for health and wellbeing in later life [24–26]. So, it is important to know how childhood obesity affects later life mental health while addressing the influence of adulthood obesity. Using genetic variants for childhood and adulthood body size and a novel multivariable Mendelian randomization (MR) method may help to answer whether childhood obesity has direct effect on later life anxiety and depression or has an indirect effect after accounting for adulthood body size [27]. MR studies use genetic variants as instrumental variable to test causal relationships between risk factors and outcomes and are typically considered to be more robust to confounding and reverse causation often seen in traditional observational studies [28, 29]. MR studies have suggested that adulthood adiposity may be a causal risk factor for later life anxiety and depression [30–36]. However, no MR study to date has investigated the causal effect of childhood adiposity on later life anxiety and depression. Hence, in this study, we aim to estimate total and direct effect (i.e., after accounting for adulthood body size) of childhood adiposity on later life anxiety and depression.

## Methods

### Study design

**Fig. 1:**
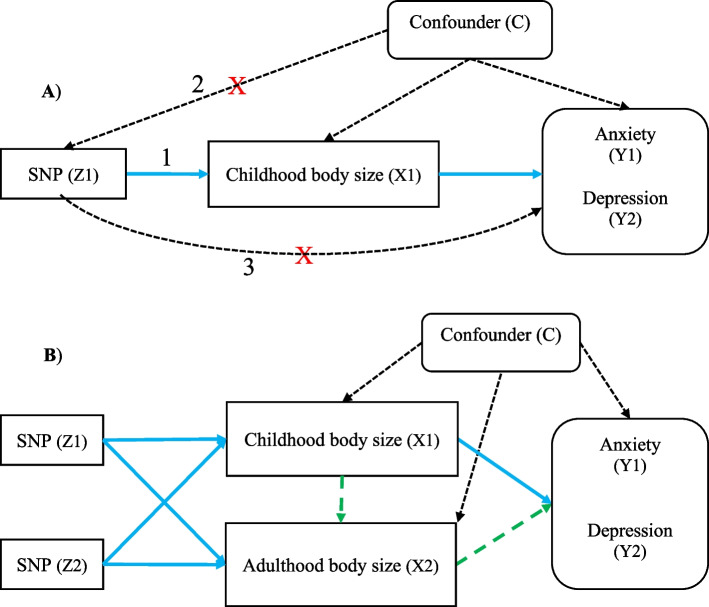
A directed acyclic graph of the two-sample MR framework. **A** Univariable MR framework in the relationship (total effect) of childhood body size (X1) on later life anxiety (Y1) and depression (Y2). The three core MR assumptions [28] are (1) the relevance—the SNP (Z1) must be associated with the exposure (X1), (2) the independence—the SNP (Z1) must not be associated with confounders of the exposure (X1)—outcome (Y1, Y2) relationship, and (3) the exclusion-restriction—the SNP (Z1) must only be associated with the outcome (Y1, Y2) via the exposure (X1). **B** Multivariable MR framework where the direct effect pathway is the solid blue line from childhood body size (X1) on later life anxiety (Y1) and depression (Y2), while the total effect pathway includes both the solid blue line from childhood (X1) and the dotted green line from adulthood body size (X2) on later life anxiety (Y1) and depression (Y2). Single nucleotide polymorphism (SNP) Z1—genetic variants associated with the exposure of interest (childhood body size), Z2—genetic variants associated with the second exposure (adulthood body size), X1—exposure of interest (childhood body size), X2—second exposure (adulthood body size), Y1 (anxiety) and Y2 (depression)—outcome

**Fig. 2:**
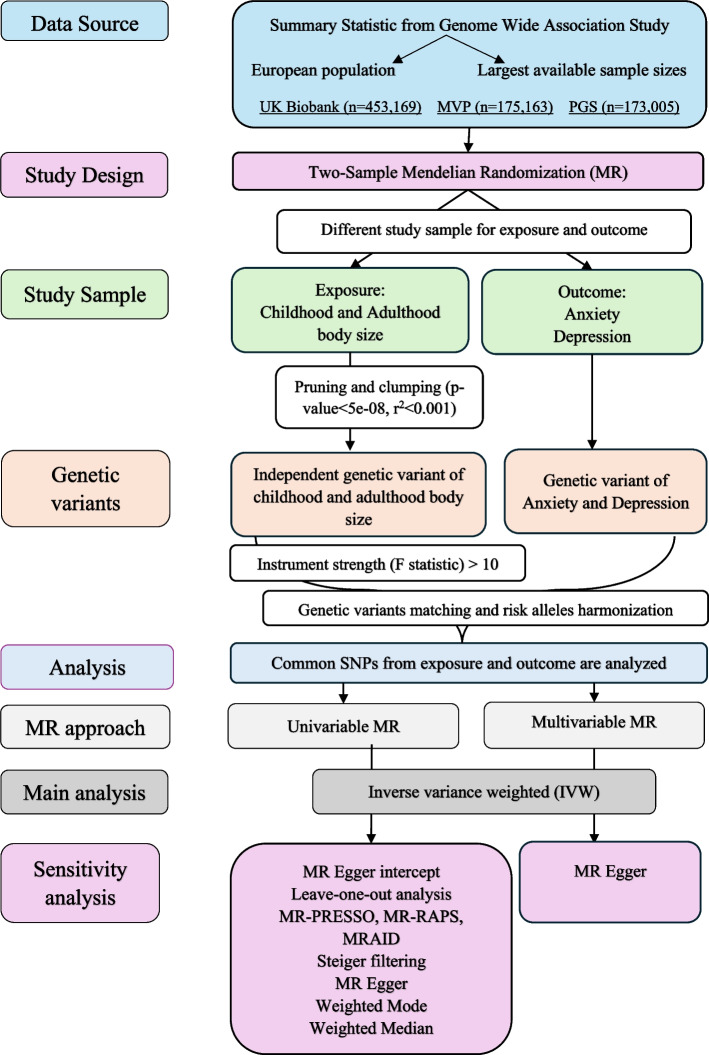
Study flow chart. Abbreviations: MVP, Million Veteran Program; PGS, Psychiatric Genomics Consortium; MR-PRESSO, MR-Pleiotropy RESidual Sum and Outlier; MR-RAPS, MR-Robust Adjusted Profile Score; MRAID, MR with automated instrument determination

We used a two-sample univariable and multivariable MR approach using summary-level data of genetic variants of childhood body size, adulthood body size, anxiety, and depression [37] (Fig. 1). We have presented a flow chart to explain potential mechanism of two-sample MR in Fig. 2.

### Data resources

#### Genetic instruments for childhood and adulthood body size

We retrieved SNP-exposure associations of childhood and adulthood body size from a genome-wide association study (GWAS) performed in UK Biobank. In this study, adult body mass index (BMI) was calculated by using height (measured in whole centimeters) and weight (to the nearest 0.1 kg) measured among people aged 40 to 69 years and at the same time people were asked to describe their childhood body size when they were 10 years as thinner, plumper, and about average [23]. To make both measures comparable, adult body size was also classified as thinner, plumper, and about average by categorizing individuals into those groups in the same proportions as was observed in the childhood data [23]. GWAS on childhood and adulthood body size among 453,169 people with European ancestry adjusted for age, sex, genotyping chip, relatedness, and population stratification was performed using linear mixed model implemented in BOLT-LMM software [23]. Conducting a bivariate GREML analysis using GCTA found that childhood and adulthood body size had a genetic correlation of rG = 0.61 [23]. In this study, 313 and 580 independent genetic variants/SNPs were identified as genetic instrument for childhood and adult body size, respectively [38]. To identify independent SNPs, a reference panel including the 10,000 randomly selected individuals of European ancestry from UK Biobank [38] was used and clumping (*r*^2^ < 0.001 and *p* value < 5e − 8) was performed. A further three validation studies were performed by using measured BMI data from independent studies in the Avon Longitudinal Study of Parents and Children (ALSPAC) [30], the Young Finns study [32], and the Trøndelag Health (HUNT) study [33] which showed that the genetic instrument for childhood body size from UK Biobank was found to be a strong predictor of childhood BMI compared to the genetic instrument for adulthood BMI. The findings were validated by calculating the risk predictive ability of genetic score of childhood and adulthood genetic instruments. Furthermore, the genetic instrument of childhood body size was distinct from the genetic instrument of adulthood body size where strong genetic correlation of childhood body size with childhood obesity (rg = 0.85) was observed compared adult BMI (rg = 0.64). Similarly, genetic correlation of adult body size with adult BMI (rg = 0.96) was much higher than with childhood body size (rg = 0.67) [23]. Details of the GWAS on childhood and adulthood body size can be found elsewhere [38]. In this study, we used both body size and body mass index measurements from UK Biobank which are proxy for adiposity [39].

#### Anxiety and depression

The summary statistics for the SNP-outcome associations (anxiety and depression) were retrieved from GWAS performed in large studies based on European ancestry.

The GWAS of anxiety was performed in the Million Veteran Program (MVP) among 175,163 people with mean age 66.58 years [40]. A linear regression model implemented in PLINK 2.0 was used to perform GWAS, which was adjusted for age, sex, and the first 10 within ancestry principle component. The GWAS of anxiety had a genomic inflation factor (*λ*) of 1.19, Linkage Disequilibrium (LD) intercept of 1.026, and the SNP heritability (*h*^2^) of 8.79%. In the study, anxiety was measured using two questionnaires (Additional file 1: Table S1), later responses were summed and then scored on a continuous scale from 0 to 6 (based on score on the Generalized Anxiety Disorder 2-item scale [GAD-2]) [9]. Details of the GWAS on anxiety can be found elsewhere [40].

The GWAS meta-analysis of depression was performed in 173,005 people (59,851 cases and 113,154 control, European ancestry) from Psychiatric Genomics Consortium (PGC) including 29 cohorts and 5 additional independent cohorts [31]. All the cohorts in the GWAS were included based on genetic and phenotypic similarity. Further, comparability of these cohorts was examined by genetic correlation of common variants between them, where rg was 0.76. The GWAS of depression had an LD intercept of 1.018 and the SNP heritability (*h*^2^) of 8.7%. Major depression cases were assessed using traditional method [Diagnostic and Statistical Manual of Mental Disorders (DSM-IV) or International Classification of Disease (ICD-9, ICD-10)] and from treatment registers, which was measured on dichotomous scale. Details of the GWAS on depression can be found elsewhere [31].

### Statistical analysis

We used univariable MR to estimate the total effect of genetically predicted childhood and adult body size on anxiety and depression. We used multivariable MR to estimate the direct effect of genetically predicted childhood body size conditioning on adult body size and vice versa on anxiety and depression. The “MendelianRandomization” package in R statistical software was used. In both univariable and multivariable analyses, inverse variance weighted (IVW) method was used as a primary analysis to estimate the causal association of the exposure on outcome. IVW estimates are obtained by combining the ratio estimates of each individual genetic variant, such ratio estimates are obtained by dividing the SNP-outcome association by the SNP-exposure association, where the weight of each ratio is the inverse of the variance [41]. If all the genetic variants used in MR are valid, the IVW estimate is most powerful [28, 42]. Furthermore, MR assumptions (Fig. 1) were thoroughly tested in our study.

The risk alleles of childhood body size were harmonized with the risk alleles of adulthood body size and outcome (anxiety and depression). Finally a harmonized data set that contain the same allele pairs were analyzed. We calculated conditional *F*-statistic to test the strength of genetic variants of childhood and adulthood body size (Additional file 1: Table S2) [43]. Valid instruments can provide independent and unbiased causal estimates of the effect of the exposure on the outcome [44].

As a sensitivity analysis to evaluate the validity of IVW results in the univariable and multivariable MR analysis, we calculated MR Egger intercepts and estimates, and additionally for the univariable MR analysis, weighted mode and weighted median, leave-one-out analyses, MR-Pleiotropy RESidual Sum and Outlier (MR-PRESSO) [45], MR-Robust Adjusted Profile Score (MR-RAPS) [46], MR with automated instrument determination (MRAID) [47], and Steiger filtering were performed to explore potential horizontal pleiotropy (that is when genetic variants for the exposure of interest has an effect on outcome through a phenotype other than the exposure).

**Fig. 3:**
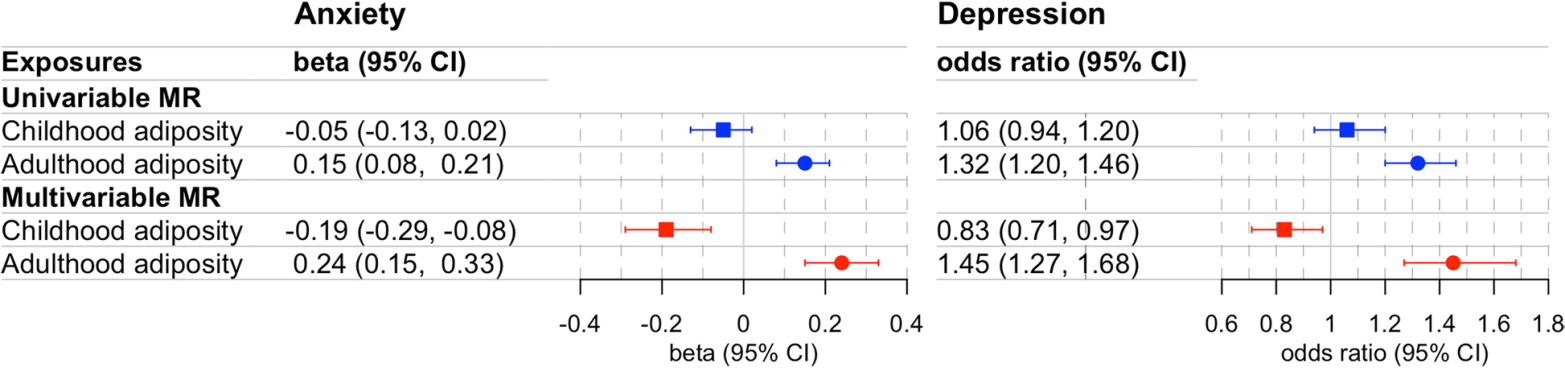
Forest plot illustrating the total and direct causal estimates of childhood adiposity and adulthood adiposity on anxiety and depression. The univariable MR represents the total causal estimate and multivariable MR represents direct causal estimates. The causal estimates are presented as beta for anxiety and odds ratio for depression with 95% CI. Abbreviations: MR, Mendelian randomization; CI, confidence interval

We presented the estimates in forest plots (Fig. 3) using the “forestplot” package in R.

## Results

### Total effects of childhood and adulthood body size on anxiety and depression

In univariable MR analysis, there was no evidence of a total effect of genetically predicted childhood adiposity on later life anxiety score (beta = − 0.05 per change in body size, 95% confidence interval (CI) = − 0.13, 0.02) and depression (odds ratio (OR) = 1.06 per change in body size, 95% CI = 0.94, 1.20), whereas, for genetically predicted adulthood adiposity, there was strong evidence of a positive effect on anxiety (beta = 0.15 per change in body size, 95% CI = 0.08, 0.21) and depression (OR = 1.32 per change in body size, 95% CI = 1.20, 1.46) (Fig. 3, Additional file 1: Table S3).

### Direct effects of childhood and adulthood body size on anxiety and depression

In multivariable MR analysis, there was evidence of a direct effect of childhood adiposity (accounting for adulthood adiposity) with a strong protective effect on anxiety (beta = − 0.19 per change in body size, 95% CI = − 0.29, − 0.08) and depression (OR = 0.83 per change in body size, 95% CI = 0.71, 0.97). Moreover, the direct effect of adulthood body size (accounting for childhood body size) was consistent with univariable MR with a strong increase in anxiety (beta = 0.24 per change in body size, 95% CI = 0.15, 0.33) and depression (OR = 1.45 per change in body size, 95% CI = 1.27, 1.68) (Fig. 3).

### Sensitivity analysis

In univariable MR, the MR Egger intercept provided no evidence of horizontal pleiotropy as the intercept was close to zero (Additional file 1: Table S4). However, the MR Egger, weighted median, and weighted mode estimates slightly varied from IVW estimates (Additional file 1: Tables S5–S6). In MR-RAPS and MRAID analysis, effect estimates were similar to IVW estimates (Additional file 1: Tables S7–S8). In leave-one-out analysis where re-estimation of the causal effect is performed by removing each genetic variant from the analysis, the results did not indicate any significant effect of any particular variant on the overall estimation. This was further tested by the MR-PRESSO analysis where no outlying SNPs were detected (Additional file 1: Fig. S1). In multivariable MR, MR Egger intercept provided no evidence of horizontal pleiotropy and MR Egger estimates were similar to IVW estimates (Additional file 1: Tables S4–S5).

We conducted Steiger filtering for all SNPs of childhood and adulthood body size (Additional file 1: Table S9, Additional file 2: Sheets 8–11). All the SNPs satisfy the directionality test which indicates that the genetic variant explains more variance in the exposure than the outcome. The conditional *F*-statistics of childhood body size conditioned on adulthood body size was > 10 suggesting that the level of bias due to weak instruments was likely to be low (Additional file 1: Table S2).

Statistical evidence of heterogeneity was observed across all analyses (Additional file 1: Table S10).

## Discussion

In this MR study, we examined the influence of early life body size (age 10 year) on later life anxiety and depression. Our multivariable MR estimates suggest that genetically predicted larger body size at childhood has a direct effect reducing the risk of later life anxiety and depression, provided that people do not continue to be obese as adults. However, higher body size in adulthood was a risk factor for later life anxiety and depression.

To our knowledge, no study has investigated the association of childhood adiposity on later life anxiety. However, Lindberg et al. observed a positive association between childhood adiposity and childhood anxiety in a nationwide Swedish study [7]. In contrast, a systematic review and meta-analysis by Moradi et al. observed no association between childhood adiposity and childhood anxiety [48]. Several other studies have suggested inconsistent associations between childhood obesity and childhood anxiety, but no studies have reported a protective association [49–51]. Therefore, further validation of our findings in an independent study is warranted.

Only two observational studies have examined the association of childhood adiposity on later life depression. Tyrrell et al. (*n* = 287,503) studied the UK Biobank population and observed that the thinner and plumper childen has increased risk of depression compared to child with average body size [21]. Gibson-Smith et al. (*n* = 889) observed increased risk of major depressive disorder (MDD) among obese childen compared to childen with normal body size; however, did not observed any association between childhood obesity and later life depressive symptoms [52]. In both of studies by Tyrrell et al. and Gibson-Smith et al., the effect of childhood adiposity remained the same even after adjusting for adulthood BMI [21, 52]. In contrast to these findings from observational studies, our study suggests a protective effect of childhood body size on later life depression after adjusting for adulthood body size using a multivariable MR approach. A possible reason for the differences in results might be due to the differences in study design, where we used a MR approach, which may have avoided residual confounding which commonly affects observational studies [28].

Besides our study, no other multivariable MR studies have examined the role of childhood body size adjusting for adulthood body size on risk of later life anxiety and depression. However, several multivariable MR studies have investigated the effect of genetically predicted childhood body size adjusting for adulthood body size on different later life health outcomes. Larger body size during childhood has been observed to increase the risk of health outcomes such as coronary artery disease, type 1 diabetes, colorectal cancer, cardiovascular disease, and heart structure [23, 53–55]. However, protective effects of genetically predicted higher body size during childhood have been observed for fracture and breast cancer in later life [23, 56]. A similar protective effect for mental health (anxiety and depression) was observed by our study for childhood adiposity, while an increased risk for poor mental health was observed for adulthood body size. These findings suggest that larger body size in childhood is not contributing to later life poor mental health, but it is large body size in adulthood that is the risk factor. Additionally, the findings highlight the public health importance of a life course approach in our understanding of adverse health outcomes, and how the quantification of risk/benefits of adiposity at different stages of life could guide targeted life course intervention and improve diseases prevention. However, having large body size during adulthood increases the risk of poor mental health.

A strength of our study is that the two-sample MR framework allowed us to use genetic instruments from GWAS with the largest available sample sizes, thus improving statistical power. In addition, in the two-sample MR approach, we used mostly non-overlapping data sets for the exposure and outcome which helps to reduce potential bias from overfitting [57]. This study used the novel multivariable MR approach to quantify the life course effect of childhood body size on later life mental health, which has used genetic variants for body size at two specific time periods over the life course [58–60]. This novel approach allowed us to separate out effects from the childhood and adulthood SNPs and therefore capture the effect of childhood adiposity on mental health after accounting for effect of adulthood adiposity.

However, the results should be interpreted in light of certain limitations. The UK Biobank contribute 17% of the total sample size in the depression GWAS used for our analysis, which is a small contribution to the overall GWAS estimates but might introduce biases towards the observational estimates in our two-sample MR analysis. The childhood body size is self-reported body size, where adult participants (average age 56.5) recalled their childhood body size when they were 10 year old. This might have introduced some recall bias. Although simulations showed that this was unlikely [23]. Additionally, as describe earlier, three validation studies in different cohorts were conducted confirming the genetic instrument for perceived childhood body size [23, 61, 62]. We cannot however rule out that the underlying pathophysiology of childhood and adulthood adiposity is not the same and therefore may reflect different pathways to disease which may complicate our interpretation. The anxiety summary statistics was derived from MVP, which may not represent the general population and could limit the generalizability of results. However, on request from the reviewers, we conducted a post hoc analysis using SNP-outcome associations for anxiety from FinnGen biobank and similar results were observed (Additional file 1: Table S11). Our univariable and multivariable MR results are also vulnerable to confounding due to population stratification, dynastic effects, and assortative mating [42]. Sex-specific analysis was not possible in this study due to the unavailability of sex-stratified GWAS summary statistics of outcomes at this time point, but should be a focus of future investigations. A further limitation of our study is the lack of ancestral diversity which limits the generalizability of results. Therefore, future research is warranted with different ancestries. Moreover, the non-linear attribute of adiposity was not taken into account and we only estimate the average causal effect. Finally, compared to the general population, UK Biobank participants are more likely to be older, female, and live in less socioeconomically deprived areas which might have introduced selection bias in our study [63].

## Conclusions

Using multivariable Mendelian randomization, our findings provide novel evidence that higher body size during childhood (accounting for adulthood body size) does not appear to be a risk factor for later life anxiety and depression, provided that normal body size during adulthood is maintained. In contrast, having larger body size during adulthood (after accounting for childhood body size) may increase the risk of anxiety and depression.

## Supplementary Information

Additional file 1: Tables S1–-S11. Table S1 GAD-2 phenotype. Table S2 *F*- statistics. Table S3 Inverse variance weighted (IVW) estimates. Table S4 MR Egger intercepts. Table S5 MR Egger estimates. Table S6 Weighted median and weighted mode estimates. Table S7 MR-Robust Adjusted Profile Score (MR-RAPS) estimates. Table S8 MR with automated instrument determination (MRAID) estimates. Fig. S1 Leave-one-out analysis plot. Table S9 Directionality test. Table S10 Heterogeneity test. Table S11 Inverse variance weighted (IVW) estimates from FinnGen biobank based anxiety GWAS

Additional file 2: Sheets 1–11

Additional file 3.

## Data Availability

The data supporting the findings are available in Additional file 2 and upon request. GWAS description and summary statistic data of childhood and adulthood body size are available in Additional file 2: Sheets 1–7. GWAS summary statistic of anxiety can be accessed through application at https://www.ncbi.nlm.nih.gov/gap/, dbGaP Study Accession phs001672. GWAS summary statistic of depression is publicly available on the Psychiatric Genomics Consortium web site: https://pgc.unc.edu/.

## Abbreviations

MR: Mendelian randomization
SNP: Single nucleotide polymorphism
GWAS: Genome-wide association study
BMI: Body mass index
IVW: Inverse variance weighted
OR: Odds ratio
CI: Confidence interval
MVP: Million Veteran Program
LD: Linkage Disequilibrium
MR-RAPS: MR-Robust Adjusted Profile Score
MRAID: MR with automated instrument determination
MR-PRESSO: MR-Pleiotropy RESidual Sum and Outlier
