# Supplementary material for: Investigating the causal effects of childhood and adulthood adiposity on later life mental health outcome: a Mendelian randomization study
Source: BMC Med. 2025 Jan 6;23:4. doi: 10.1186/s12916-024-03765-6 (PMC11702221; doi:10.1186/s12916-024-03765-6)
Supplement: Supplementary file 1 — Additional file 1: Tables S1–-S11. Table S1 GAD-2 phenotype. Table S2 F- statistics. Table S3 Inverse variance weighted (IVW) estimates. Table S4 MR Egger intercepts. Table S5 MR Egger estimates. Table S6 Weighted median and weighted mode estimates. Table S7 MR-Robust Adjusted Profile Score (MR-RAPS) estimates. Table S8 MR with automated instrument determination (MRAID) estimates. Fig. S1 Leave-one-out analysis plot. Table S9 Directionality test. Table S10 Heterogeneity test. Table S11 Inverse variance weighted (IVW) estimates from FinnGen biobank based anxiety GWAS [file 12916_2024_3765_MOESM1_ESM.docx]

**Investigating the causal effects of childhood and adulthood adiposity on later life mental health outcome: a Mendelian randomization study**

Sweta Pathak^1^ *, Tom G Richardson^2^, Eleanor Sanderson^2^, Bjørn Olav Åsvold^1,3,4^, Laxmi Bhatta^1 #^, Ben M. Brumpton^1,4,5 #^*

^1^ HUNT Center for Molecular and Clinical Epidemiology, Department of Public Health and Nursing, Norwegian University of Science and Technology, Trondheim, Norway

^2^ MRC Integrative Epidemiology Unit, Population Health Sciences, Bristol Medical School, University of Bristol, United Kingdom

^3^ Department of Endocrinology, Clinic of Medicine, St. Olavs Hospital, Trondheim University Hospital, Trondheim 7030, Norway.

^4^ HUNT Research Centre, Department of Public Health and Nursing, NTNU Norwegian University of Science and Technology, Levanger, Norway.

^5^ Clinic of Medicine, St. Olavs Hospital, Trondheim University Hospital, Trondheim, Norway.

**^#^ Equal contributions**

* **Corresponding authors**:

Sweta Pathak [sweta.pathak@ntnu.no](mailto:sweta.pathak@ntnu.no), Ben Brumpton [ben.brumpton@ntnu.no](mailto:ben.brumpton@ntnu.no)

Department of Public Health and Nursing, Faculty of Medicine and Health Sciences, NTNU Norwegian University of Science and Technology, P.O. Box 8905, MTFS, NO-7491, Trondheim, Norway

**Table S1.** GAD-2 Phenotype.

| **GAD-2** | | | | |
| --- | --- | --- | --- | --- |
| Over the last 2 weeks, how often have you been bothered by the following problems ?  (Use “√” to indicate your answer) | Not at all | Several days | More than half the days | Nearly every day |
| Feeling nervous, anxious, or on edge | 0 | 1 | 2 | 3 |
| Not being able to stop or control worrying | 0 | 1 | 2 | 3 |

**Table S2**. F statistics in univariable and conditional F statistics in multivariable MR.

| **Exposure** | **Outcome** | **Univariable MR** | **Multivariable MR** |
| --- | --- | --- | --- |
| Childhood adiposity | Anxiety | 65.35 | 13.34 |
|  | Depression | 66.03 | 13.47 |
| Adulthood adiposity | Anxiety | 52.14 | 15.74 |
|  | Depression | 52.43 | 15.60 |

Abbreviations: MR, Mendelian randomization

**Table S3.** Univariable and multivariable Inverse Variance Weighted (IVW) estimates of childhood and adulthood adiposity on anxiety and depression.

| **Exposures** | **Outcomes** | **Univariable MR** | | | | **Multivariable MR** | | | |
| --- | --- | --- | --- | --- | --- | --- | --- | --- | --- |
|  |  | **SNPs** | **Beta/OR*** | **95% CI** | **P-value** | **SNPs** | **Beta/OR*** | **95% CI** | **P-value** |
| Childhood adiposity | Anxiety | 277 | -0.05 | -0.13 to 0.02 | 0.171 | 652 | -0.19 | -0.29 to -0.08 | 3.86×10^-04^ |
|  | Depression | 260 | 1.06 | 0.94 to 1.20 | 0.345 | 638 | 0.83 | 0.71 to 0.97 | 0.019 |
| Adulthood adiposity | Anxiety | 510 | 0.15 | 0.08 to 0.21 | 1.11×10^-05^ | 652 | 0.24 | 0.15 to 0.33 | 1.09×10^-07^ |
|  | Depression | 496 | 1.32 | 1.20 to 1.46 | 3.31×10^-08^ | 638 | 1.45 | 1.27 to 1.68 | 1.33×10^-07^ |

Table illustrating the total (univariable MR) and direct (multivariable MR) causal estimates of childhood and adulthood adiposity on later life anxiety and depression.

*The causal estimates are presented as beta for anxiety and odds ratio for depression with 95% CI and P-value. Number of SNPs (genetic variant) involve in analysis to investigate causal effect of exposure on outcome also presented.

Abbreviations: MR, Mendelian randomization; CI, confidence interval; SNP, Single nucleotide polymorphism; OR, Odds ratio

**Table S4.** Univariable and multivariable MR Egger intercepts of childhood and adulthood adiposity on anxiety and depression.

| **Exposures** | **Outcomes** | **Univariable MR** | | | **Multivariable MR** | | |
| --- | --- | --- | --- | --- | --- | --- | --- |
|  |  | **SNPs** | **intercept** | **P-value** | **SNPs** | **intercept** | **P-value** |
| Childhood adiposity | Anxiety | 277 | -0.0002 | 0.784 | 652 | 0.000 | 0.583 |
|  | Depression | 260 | -0.002 | 0.367 | 638 | -0.001 | 0.432 |
| Adulthood adiposity | Anxiety | 510 | 0.002 | 0.142 | 652 | 0.001 | 0.583 |
|  | Depression | 496 | 0.002 | 0.185 | 638 | -0.001 | 0.432 |

Abbreviations: MR, Mendelian randomization; SNP, Single nucleotide polymorphism

**Table S5.** Univariable and multivariable MR Egger estimates of childhood and adulthood adiposity on anxiety and depression.

| **Exposures** | **Outcomes** | **Univariable MR** | | | | **Multivariable MR** | | | |
| --- | --- | --- | --- | --- | --- | --- | --- | --- | --- |
|  |  | **SNPs** | **Beta/OR** | **95% CI** | **P-value** | **SNPs** | **Beta/OR** | **95% CI** | **P-value** |
| Childhood adiposity | Anxiety | 277 | -0.03 | -0.19 to 0.13 | 0.702 | 652 | -0.17 | -0.29 to -0.06 | 0.004 |
|  | Depression | 260 | 1.18 | 0.91 to 1.55 | 0.216 | 638 | 0.85 | 0.71 to 1.02 | 0.085 |
| Adulthood adiposity | Anxiety | 510 | 0.02 | -0.17 to 0.20 | 0.854 | 652 | 0.26 | 0.16 to 0.35 | 3.40×10^-07^ |
|  | Depression | 496 | 1.11 | 0.83 to 1.47 | 0.489 | 638 | 1.49 | 1.28 to 1.74 | 2.93×10^-07^ |

Abbreviations: MR, Mendelian randomization; CI, confidence interval; SNP, Single nucleotide polymorphism; OR, Odds ratio

**Table S6.** Univariable weighted median and weighted mode estimates of childhood and adulthood adiposity on anxiety and depression.

| **Exposures** | **Outcomes** | **Univariable weight median** | | | | **Univariable weighted mode** | | | |
| --- | --- | --- | --- | --- | --- | --- | --- | --- | --- |
|  |  | **SNPs** | **Beta/OR** | **95% CI** | **P-value** | **SNPs** | **Beta/OR** | **95% CI** | **P-value** |
| Childhood adiposity | Anxiety | 277 | 0.006 | -0.10 to 0.11 | 0.904 | 277 | 0.04 | -0.11 to 0.21 | 0.589 |
|  | Depression | 260 | 1.20 | 1.02 to 1.42 | 0.032 | 260 | 1.28 | 1.05 to 1.57 | 0.014 |
| Adulthood adiposity | Anxiety | 510 | 0.12 | 0.02 to 0.22 | 0.020 | 510 | 0.16 | -0.02 to 0.34 | 0.11 |
|  | Depression | 496 | 1.43 | 1.25 to 1.64 | 5.34×10^-07^ | 496 | 1.58 | 1.17 to 2.13 | 0.003 |

Abbreviations: CI, confidence interval; SNP, Single nucleotide polymorphism; OR, Odds ratio

**Table S7.** MR-Robust Adjusted Profile Score (MR-RAPS) estimates for from childhood and adulthood adiposity to anxiety and depression.

| **Exposure** | **Outcome** | **SNPs** | **Beta/OR** | **P-value** |
| --- | --- | --- | --- | --- |
| Childhood adiposity | Anxiety | 277 | -0.04 | 0.343 |
|  | Depression | 260 | 1.09 | 0.159 |
| Adulthood adiposity | Anxiety | 510 | 0.17 | <0.005 |
|  | Depression | 496 | 1.37 | <0.005 |

Abbreviations: MR, Mendelian randomization; SNP, Single nucleotide polymorphism; OR, Odds ratio

**Table S8.** MR with Automated Instrument Determination (MRAID) estimates for from childhood and adulthood adiposity to anxiety and depression.

| **Exposure** | **Outcome** | **Beta/OR** | **P-value** |
| --- | --- | --- | --- |
| Childhood adiposity | Anxiety | -0.001 | 0.971 |
|  | Depression | 0.84 | 1.213 |
| Adulthood adiposity | Anxiety | 1.36 | <0.005 |
|  | Depression | 3.29 | <0.005 |

Note: LD matrixes were derived using 1000 genome reference panel for European population. Same LD matrix were used for exposure and outcome. The instruments used for exposure were clumped on r2<0.001.

Abbreviations: OR, Odds ratio

**Supplement Figure S1.** Leave-one-out anysis plot for Inverse Variance Weighted (IVW) estimates of childhood and adulthood adiposity on anxiety and depression.

1. Childhood Adiposity on Anxiety B) Adulthood Adiposity on Anxiety


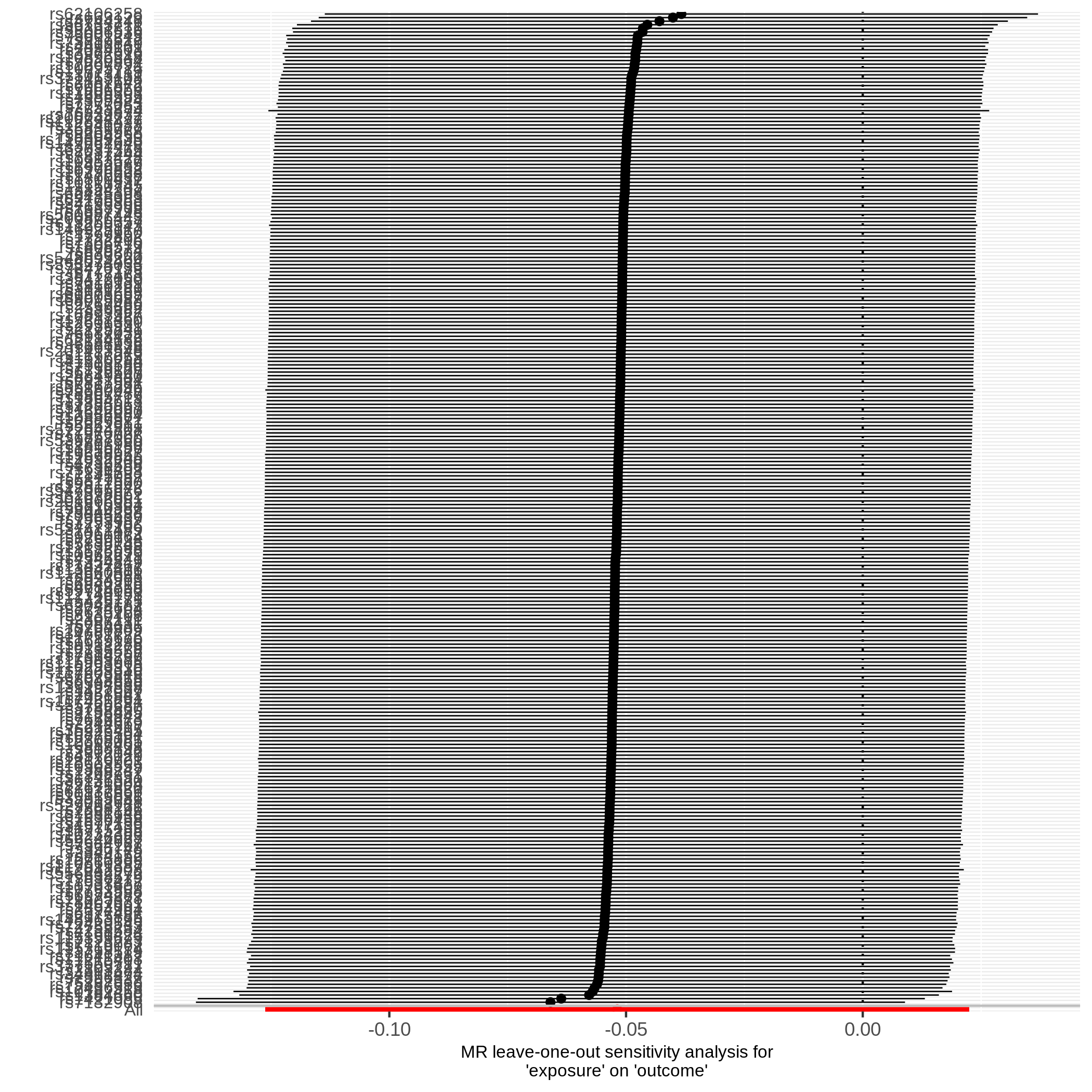

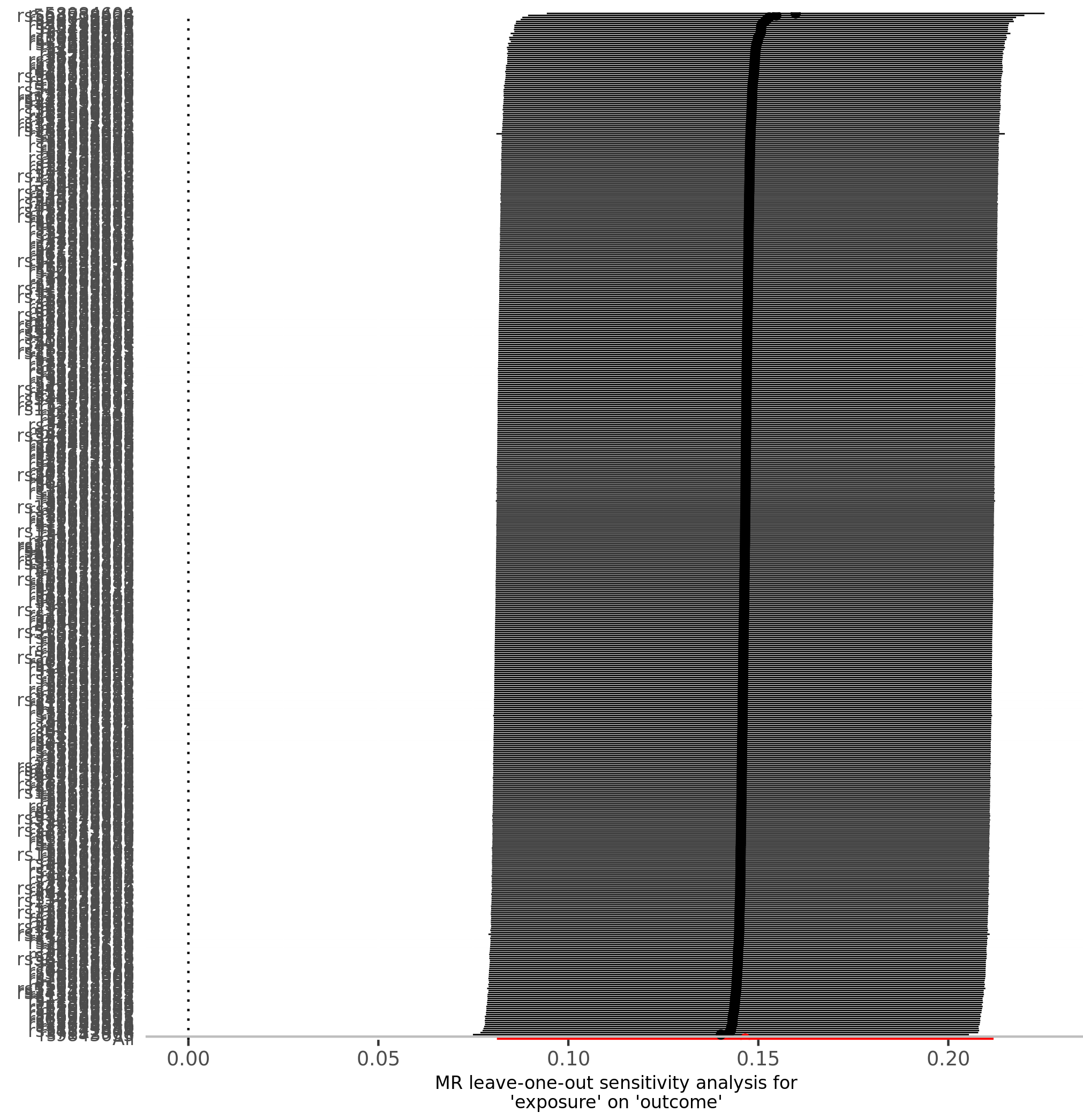


1. Childhood Adiposity on Depression D) Adulthood Adiposity on Depression


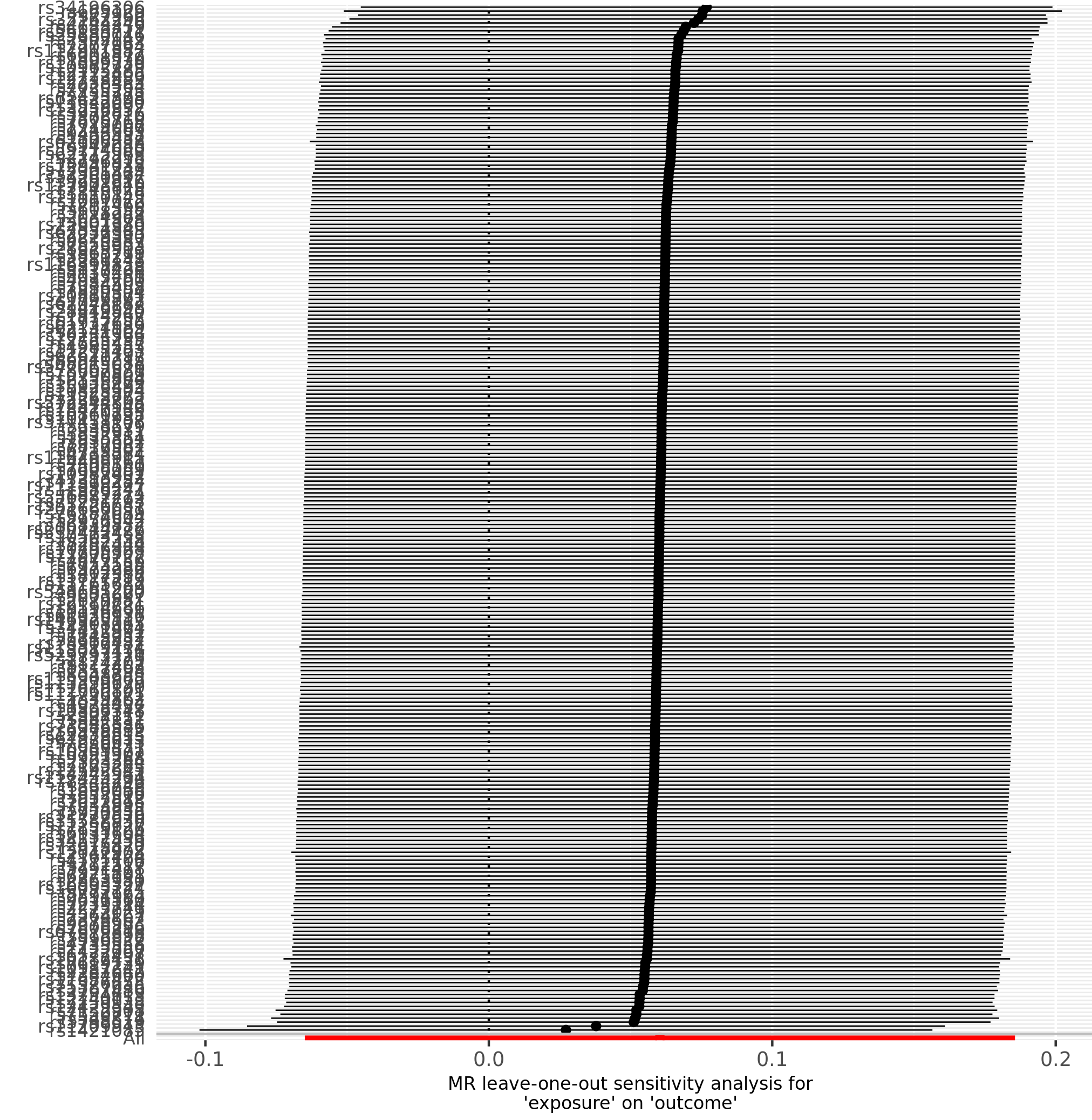

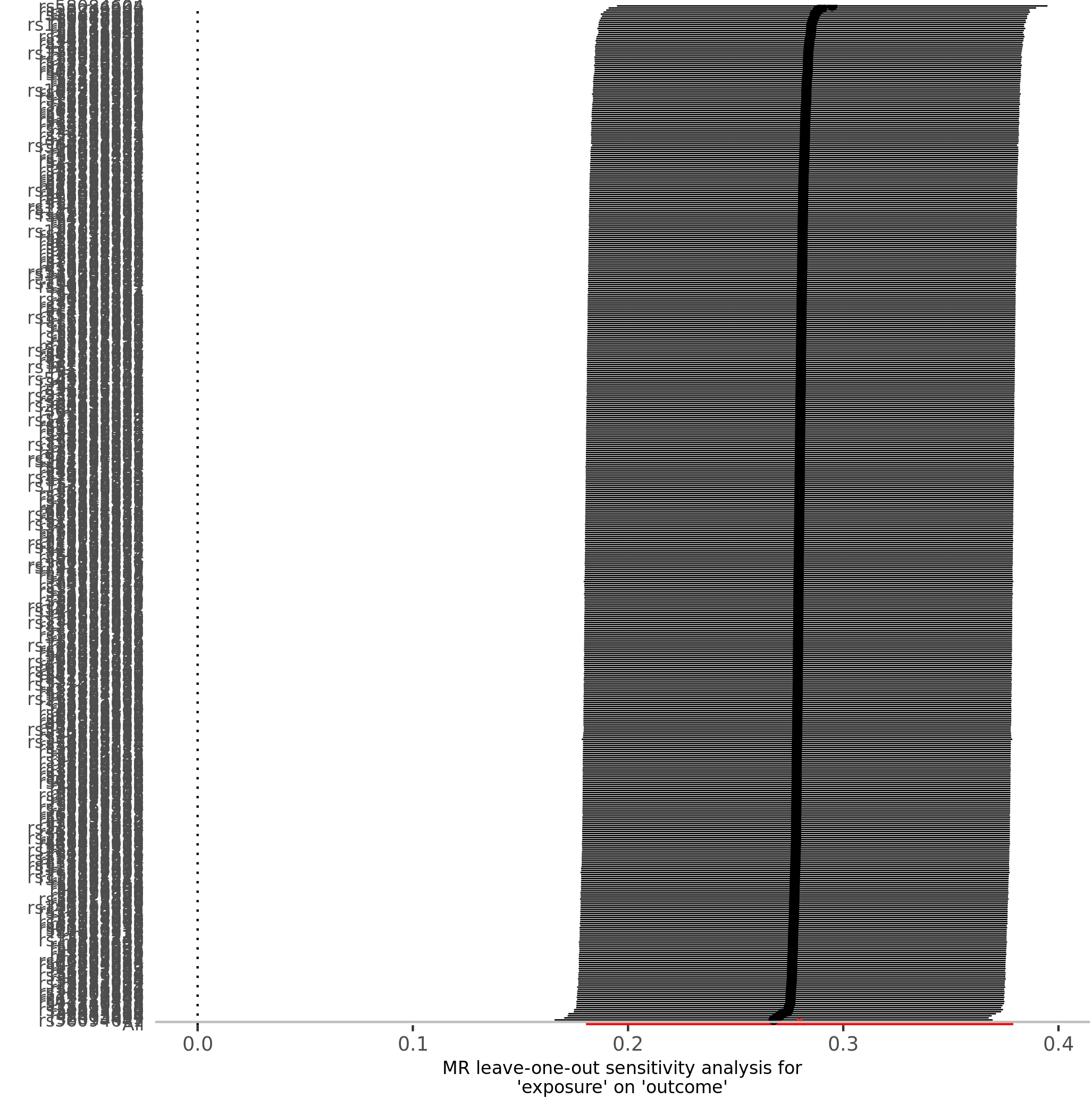


**Table S9.** Directionality test from childhood and adulthood adiposity to anxiety and depression.

| **Exposure** | **Outcome** | **snp_r2.exposure** | **snp_r2.outcome** | **correct_causal_direction** | **steiger_pval** |
| --- | --- | --- | --- | --- | --- |
| Childhood adiposity | Anxiety | 0.040 | 0.002 | True | <0.005 |
|  | Depression | 0.038 | 0.009 | True | <0.005 |
| Adulthood adiposity | Anxiety | 0.059 | 0.005 | True | <0.005 |
|  | Depression | 0.058 | 0.018 | True | <0.005 |

**Table S10**. Heterogeneity test of univariable and multivariable MR.

| **Exposure** | **Outcome** | **Univariable MR** | **Multivariable MR** |
| --- | --- | --- | --- |
|  |  | **Q (P-value)** | **Q (P-value)** |
| Childhood adiposity | Anxiety | 443 (7.34×10^-10^) | 1076 (1.46×10^-23^) |
|  | Depression | 527 (1.76×10^-20^) | 1134 (1.40×10^-30^) |
| Adulthood adiposity | Anxiety | 885 (1.05×10^-22^) | 1076 (1.46×10^-23^) |
|  | Depression | 874 (2.18×10^-23^) | 1134 (1.40×10^-30^) |

Abbreviations: MR, Mendelian randomization

**Table S11.** Univariable and multivariable Inverse Variance Weighted (IVW) estimates of childhood and adulthood adiposity on anxiety. A validation study using anxiety summary statistics from FinnGen Biobank (KRA_PSY_ANXIETY, FREEZE 11 release).

| **Exposures** | **Outcomes** | **Univariable MR** | | | | **Multivariable MR** | | | |
| --- | --- | --- | --- | --- | --- | --- | --- | --- | --- |
|  |  | **SNPs** | **OR** | **95% CI** | **P-value** | **SNPs** | **OR** | **95% CI** | **P-value** |
| Childhood adiposity | Anxiety | 248 | 0.88 | 0.78 to 0.99 | 0.030 | 621 | 0.76 | 0.66 to 0.89 | 0.0004 |
| Adulthood adiposity | Anxiety | 464 | 1.01 | 0.92 to 1.11 | 0.804 | 621 | 1.19 | 1.04 to 1.35 | 0.010 |

Table illustrating the total (univariable MR) and direct (multivariable MR) causal estimates of childhood and adulthood adiposity on later life anxiety. The anxiety summary statictics were derived from FinnGen Biobank (N_case_ = 50,486, N_control_ = 330,560). The ICD codes used to categorise anxiety were: ICD8 (3000, 3001, 3002, 30030, 3005, 3006, 3007, 3008, 3009, 305, 30680, 30799); ICD9 (3000, 3001, 3002, 3003, 3006, 3007, 3008, 3009, 3078A, 309); ICD10 (F40, F41, F42, F43, F44, F45, F46, F47, F48)

Abbreviations: MR, Mendelian randomization; CI, confidence interval; SNP, Single nucleotide polymorphism; OR, Odds ratio
