# Supplementary material for: Investigating the causal effects of childhood and adulthood adiposity on later life mental health outcome: a Mendelian randomization study
Source: BMC Med. 2025 Jan 6;23:4. doi: 10.1186/s12916-024-03765-6 (PMC11702221; doi:10.1186/s12916-024-03765-6)
Supplement: Supplementary file 3 — Additional file 3. [file 12916_2024_3765_MOESM3_ESM.docx]

**STROBE-MR checklist of recommended items to address in reports of Mendelian randomization studies**^1^ ^2^

| **Item No.** | **Section** | **Checklist item** | **Page No.** | **Relevant text from manuscript** |
| --- | --- | --- | --- | --- |
| 1 | **TITLE and ABSTRACT** | Indicate Mendelian randomization (MR) as the study’s design in the title and/or the abstract if that is a main purpose of the study | 1 | Investigating the causal effects of childhood and adulthood adiposity on later life mental health outcome: a Mendelian randomization study. |
|  | **INTRODUCTION** |  |  |  |
| 2 | **Background** | Explain the scientific background and rationale for the reported study. What is the exposure? Is a potential causal relationship between exposure and outcome plausible? Justify why MR is a helpful method to address the study question | 3-4 | It is explained in the background section. |
| 3 | **Objectives** | State specific objectives clearly, including pre-specified causal hypotheses (if any). State that MR is a method that, under specific assumptions, intends to estimate causal effects | 3-4 | We have described this in the 3^rd^ paragraph of the background section. |
|  | **METHODS** |  |  |  |
| 4 | **Study design and data sources** | Present key elements of the study design early in the article. Consider including a table listing sources of data for all phases of the study. For each data source contributing to the analysis, describe the following: | 4-6, 12 | We have used two-sample mendelian randomization approach to explore causal effect of exposure on outcome using publicly available summary-level data from genome-wide association studies (GWAS). Studies (data source) are listed in the **Additional file 2: Sheet 1** and text detail about data source are described in the manuscript. |
|  | a) | Setting: Describe the study design and the underlying population, if possible. Describe the setting, locations, and relevant dates, including periods of recruitment, exposure, follow-up, and data collection, when available. | 4-6 |  |
|  | b) | Participants: Give the eligibility criteria, and the sources and methods of selection of participants. Report the sample size, and whether any power or sample size calculations were carried out prior to the main analysis | 4-6 |  |
|  | c) | Describe measurement, quality control and selection of genetic variants | 4-6 | Described under the section data resources. |
|  | d) | For each exposure, outcome, and other relevant variables, describe methods of assessment and diagnostic criteria for diseases | 4-6 | Described under the section data resources. |
|  | e) | Provide details of ethics committee approval and participant informed consent, if relevant | 12 | We used publicly available summary statistics from published studies. Ethical approval and participant consent for each study is detailed in the respective publications. |
| 5 | **Assumptions** | Explicitly state the three core IV assumptions for the main analysis (relevance, independence and exclusion restriction) as well assumptions for any additional or sensitivity analysis | 13 | 3 IV assumptions are described in **Figure 1**. |
| 6 | **Statistical methods: main analysis** | Describe statistical methods and statistics used | 6 | All the relevant information regarding statistical analysis of the study are describe in the ´Statistical Analysis´ section. |
|  | a) | Describe how quantitative variables were handled in the analyses (i.e., scale, units, model) |  |  |
|  | b) | Describe how genetic variants were handled in the analyses and, if applicable, how their weights were selected |  |  |
|  | c) | Describe the MR estimator (e.g. two-stage least squares, Wald ratio) and related statistics. Detail the included covariates and, in case of two-sample MR, whether the same covariate set was used for adjustment in the two samples |  |  |
|  | d) | Explain how missing data were addressed |  | Not applicable |
|  | e) | If applicable, indicate how multiple testing was addressed |  | Not applicable |
| 7 | **Assessment of assumptions** | Describe any methods or prior knowledge used to assess the assumptions or justify their validity | 6 | We have mentioned about the different test to assess the validity of assumptions in the ‘Statistical Analysis’ section. |
| 8 | **Sensitivity analyses and additional analyses** | Describe any sensitivity analyses or additional analyses performed (e.g. comparison of effect estimates from different approaches, independent replication, bias analytic techniques, validation of instruments, simulations) | 6 | Different sensitivity analysis are performed which are mention in the `Statistical Analysis section. |
| 9 | **Software and pre-registration** |  |  |  |
|  | a) | Name statistical software and package(s), including version and settings used | 6 | The `MendelianRandomization` package in R statistical software (version: 4.1.2) was used. |
|  | b) | State whether the study protocol and details were pre-registered (as well as when and where) |  | Not applicable |
|  | **RESULTS** |  |  |  |
| 10 | **Descriptive data** |  |  |  |
|  | a) | Report the numbers of individuals at each stage of included studies and reasons for exclusion. Consider use of a flow diagram |  | Not applicable |
|  | b) | Report summary statistics for phenotypic exposure(s), outcome(s), and other relevant variables (e.g. means, SDs, proportions) | 7 | List of summary statistic used in this study are reported in the **Additional file 2** and detail about data availability are reported in the manuscript under `Availability of data and materials’ section. |
|  | c) | If the data sources include meta-analyses of previous studies, provide the assessments of heterogeneity across these studies |  | Not available |
|  | d) | For two-sample MR:  i.  Provide justification of the similarity of the genetic variant-exposure associations between the exposure and outcome samples  ii.  Provide information on the number of individuals who overlap between the exposure and outcome studies | 4-6  9-10 | Both samples are of European ancestry.  0% overlap between exposure and anxiety (outcome), and 17% overlap between exposure and depression (outcome). |
| 11 | **Main results** |  |  |  |
|  | a) | Report the associations between genetic variant and exposure, and between genetic variant and outcome, preferably on an interpretable scale | 7-8 | All the results are presented under the results section. |
|  | b) | Report MR estimates of the relationship between exposure and outcome, and the measures of uncertainty from the MR analysis, on an interpretable scale, such as odds ratio or relative risk per SD difference |  |  |
|  | c) | If relevant, consider translating estimates of relative risk into absolute risk for a meaningful time period |  |  |
|  | d) | Consider plots to visualize results (e.g. forest plot, scatterplot of associations between genetic variants and outcome versus between genetic variants and exposure) | 13 | Forest plot is presented in the manuscript. |
| 12 | **Assessment of assumptions** |  |  |  |
|  | a) | Report the assessment of the validity of the assumptions | 7-8 | We have conducted Steiger directionality test for all the SNPs as well as for the overall SNPs of childhood and adulthood body size (**Additional file 1: Table S9**) (**Additional file 2: Sheet 8-11**). All the SNPs satisfy the directionality which shows that genetic variant explain more variance in the exposure than the outcome. The conditional F-statistics of childhood body size conditioned on adulthood body size was >10 suggesting that the weak instruments bias was likely to be low (**Additional file 1: Table S2**). |
|  | b) | Report any additional statistics (e.g., assessments of heterogeneity across genetic variants, such as *I^2^*, Q statistic or E-value) | 8 | **Additional file 1: Table S10**. |
| 13 | **Sensitivity analyses and additional analyses** |  |  |  |
|  | a) | Report any sensitivity analyses to assess the robustness of the main results to violations of the assumptions | 7-8 | Presented in the manuscript ‘Result’ section |
|  | b) | Report results from other sensitivity analyses or additional analyses | 7-8 | Presented in the manuscript ‘Result’ section |
|  | c) | Report any assessment of direction of causal relationship (e.g., bidirectional MR) |  | Not applicable. |
|  | d) | When relevant, report and compare with estimates from non-MR analyses |  | Not applicable |
|  | e) | Consider additional plots to visualize results (e.g., leave-one-out analyses) |  | **Additional file 1: Figure S1.** |
|  | **DISCUSSION** |  |  |  |
| 14 | **Key results** | Summarize key results with reference to study objectives | 8 | 1^st^ paragraph of the discussion section. |
| 15 | **Limitations** | Discuss limitations of the study, taking into account the validity of the IV assumptions, other sources of potential bias, and imprecision. Discuss both direction and magnitude of any potential bias and any efforts to address them | 10 | Last paragraph of the discussion |
| 16 | **Interpretation** |  |  |  |
|  | a) | Meaning: Give a cautious overall interpretation of results in the context of their limitations and in comparison with other studies | 8-9 | Described in the discussion section |
|  | b) | Mechanism: Discuss underlying biological mechanisms that could drive a potential causal relationship between the investigated exposure and the outcome, and whether the gene-environment equivalence assumption is reasonable. Use causal language carefully, clarifying that IV estimates may provide causal effects only under certain assumptions | 8-9 | Described in the discussion section |
|  | c) | Clinical relevance: Discuss whether the results have clinical or public policy relevance, and to what extent they inform effect sizes of possible interventions | 8-9 | Describe in the discussion section. |
| 17 | **Generalizability** | Discuss the generalizability of the study results (a) to other populations, (b) across other exposure periods/timings, and (c) across other levels of exposure | 10 | Last paragraph of the discussion |
|  | **OTHER INFORMATION** |  |  |  |
| 18 | **Funding** | Describe sources of funding and the role of funders in the present study and, if applicable, sources of funding for the databases and original study or studies on which the present study is based | 11 | This is not a funded study. However, financial support for the author and supervisor are mentioned in the manuscript. |
| 19 | **Data and data sharing** | Provide the data used to perform all analyses or report where and how the data can be accessed, and reference these sources in the article. Provide the statistical code needed to reproduce the results in the article, or report whether the code is publicly accessible and if so, where | 11-12 | Mentioned in the availability of data and materials section. |
| 20 | **Conflicts of Interest** | All authors should declare all potential conflicts of interest | 12 | TGR is a full time employee of GlaxoSmithKline outside of this research. All other authors declare no competing interests. |

This checklist is copyrighted by the Equator Network under the Creative Commons Attribution 3.0 Unported (CC BY 3.0) license.

1. Skrivankova VW, Richmond RC, Woolf BAR, Yarmolinsky J, Davies NM, Swanson SA, et al. Strengthening the Reporting of Observational Studies in Epidemiology using Mendelian Randomization (STROBE-MR) Statement. JAMA. 2021;under review.

2. Skrivankova VW, Richmond RC, Woolf BAR, Davies NM, Swanson SA, VanderWeele TJ, et al. Strengthening the Reporting of Observational Studies in Epidemiology using Mendelian Randomisation (STROBE-MR): Explanation and Elaboration. BMJ. 2021;375:n2233.
